# Hydroxychloroquine reduces metastatic tumor burden in pancreatic adenocarcinoma through myeloperoxidase inhibition

**DOI:** 10.1186/s12950-025-00456-8

**Published:** 2025-08-12

**Authors:** Britney Niemann, Pavan Rao, Quinn Hopen, Kaitlyn Landreth, Angisha Basnet, Werner Geldenhuys, Tracy W. Liu, Brian A. Boone

**Affiliations:** 1 Department of Surgery, Division of Surgical Oncology, West Virginia University, Morgantown, WV 26506 US; 2 Department of Microbiology, Immunology and Cell Biology, West Virginia University, Morgantown, WV 26506 US; 3 Pharmaceutical Sciences, West Virginia University, Morgantown, WV 26506 US; 4 Neuroscience, West Virginia University, Morgantown, WV 26506 US; 5 West Virginia University Cancer Institute, West Virginia University, One Medical Center Drive, PO Box 9238 HSCS, Morgantown, WV 26506 US

**Keywords:** Hydroxychloroquine, Myeloperoxidase, Pancreatic adenocarcinoma, Metastasis, Reactive oxygen species

## Abstract

**Background:**

Metastatic pancreatic adenocarcinoma has a 5-year survival of only 3%. Neutrophil extracellular traps are formed when neutrophils expel their intracellular contents and have been intricately linked to metastases. Hydroxychloroquine is an FDA-approved anti-malarial drug and neutrophil extracellular trap inhibitor with high potential for clinical translation. This study investigates the impact of hydroxychloroquine treatment on pancreatic metastases.

**Results:**

Hydroxychloroquine reduced metastatic tumor burden via a neutrophil extracellular trap independent mechanism and resulted in prolonged survival. Hydroxychloroquine inhibited the function of myeloperoxidase in vitro via direct binding with a Kd of 9.74 mM. Myeloperoxidase inhibition via hydroxychloroquine in vivo was the direct result of suppressed activity. Hydroxychloroquine mediated myeloperoxidase inhibition was also demonstrated in metastatic pancreatic adenocarcinoma patients receiving neoadjuvant chemotherapy.

**Conclusion:**

Hydroxychloroquine suppressed pancreatic metastases growth through myeloperoxidase inhibition, leading to a significant increase in survival. Corroborative data supports this mechanism in metastatic pancreatic adenocarcinoma patients treated with hydroxychloroquine. These data provide important insight into the role of myeloperoxidase in pancreatic metastases and the potential use of hydroxychloroquine in metastatic pancreatic adenocarcinoma treatment.

**Supplementary Information:**

The online version contains supplementary material available at 10.1186/s12950-025-00456-8.

## Background

Pancreatic adenocarcinoma (PDAC) is the third leading cause of cancer-related death in the United States with a five-year survival of only 13% [1]. This is largely due to the aggressive nature of this disease, which leads to early metastases, most commonly to the liver [2]. Unfortunately, 51% of patients already have metastatic disease at the time of initial diagnosis, making them ineligible for surgical resection [3]. The only treatment option for these patients is systemic chemotherapy, which has a five-year survival of only 3% [3, 4]. While surgery is the only potentially curative treatment, many eligible patients likely have undetectable micrometastases at the time of diagnosis that cannot be identified on imaging [5–7]. Systemic therapy, most commonly FOLFIRINOX, is important to address these micrometastases and has been associated with prolonged median overall survival (OS) [4]. However, FOLFIRINOX can have poor tolerability leading to decreased chemotherapy completion rates [4, 8]. Furthermore, despite treatment, many patients develop recurrence within two years, likely related to systemic micrometastases. This highlights the urgent need for more effective systemic therapies for PDAC to improve patient outcomes [4, 9, 10].

The development of efficacious systemic therapies for PDAC patients requires an improved understanding of PDAC tumor biology involved in treatment resistance and formation of metastasis. Neutrophil extracellular traps (NETs) appear to be a key mediator in both of these processes. NETs were first described in the setting of infection where activated neutrophils released their intracellular contents through a process mediated by peptidyl arginine deiminase 4 (PAD4) [11–13]. PAD4 citrullinates histones, resulting in the unwinding and subsequent expulsion of the neutrophil’s DNA [11, 12]. NETs have been implicated in multiple types and stages of malignancies, including PDAC [14–19]. NETs consist of a matrix of DNA, proteins, and granular contents such as myeloperoxidase (MPO) and neutrophil elastase (NE), which are common markers for NETs. MPO also contributes to NET formation via disintegration of the nuclear envelope and enhancement of chromatin decondensation [20, 21]. MPO can be cytotoxic as a result of its production of reactive oxygen species (ROS). This is beneficial in the setting of infection but can also be detrimental in cancer, leading to carcinogenesis secondary to DNA damage, immunosuppression, tumor progression, and metastases [22, 23]. At the primary tumor site, NETs remodel the extracellular matrix, facilitating treatment resistance by restricting chemotherapy delivery and enhancing cancer cell migration and metastasis. NETs can also entrap cancer cells leading to dissemination [24]. In PDAC in particular, NETs contribute to the growth of primary and metastatic tumors, and their presence is associated with worse survival for patients [19]. As a result, NETs are a promising target to enhance the effectiveness of systemic therapy and improve patient outcomes.

Hydroxychloroquine (HCQ) is an FDA approved, orally bioavailable 4-amioquinoline with a low side-effect profile. It has been used since the 1940’s to treat malaria and later viral infections and autoimmune disorders [25]. Due to its established safety profile, oral bioavailability, and low cost, the use of HCQ in other pathological states has been an active focus of research [25–29]. A multitude of clinical applications for HCQ have been found due to its diverse mechanisms of action. These mechanisms include the inhibition of autophagy, blockade of toll-like receptors, and prevention of thrombosis [30]. HCQ also inhibits peptidyl arginine deiminase 4 (PAD4) [31], an enzyme important in NET formation. PAD4 exchanges arginine for citrulline on histones, allowing for unwinding of DNA and subsequent expulsion from the neutrophil during NET formation. Therapeutic or genetic inhibition of PAD4 results in substantially decreased NET production [32, 33]. Multiple pre-clinical studies and clinical trials in the neoadjuvant setting have found HCQ has anti-neoplastic effects in PDAC [34–39]. A trial evaluating HCQ in advanced metastatic disease showed no improvement in survival, although improve treatment response rate was noted when given in combination with gemcitabine and nab-paclitaxel [40]. However, given the important role of NETs in the formation of metastatic disease and the NET inhibiting function of HCQ, we investigated whether administration of HCQ would prevent early PDAC metastases in murine models.

## Methods

### Mouse strains

Two mouse strains were utilized including wild-type (WT) C57Bl/J6 mice and PAD4 knockout (PAD4-/-) mice, both of which were purchased from Jackson laboratories. Prior to any animal experiments, approval was obtained via the Institutional Animal Care and Use Committee of West Virginia University (Protocol # 1809018204). All experiments were performed according to the rules from the office of laboratory animal services of West Virginia University.

### Treatments

Hydroxychloroquine sulfate (Fisher Scientific, #747-36-4) was dissolved in water at 0.5 mg/mL. Mice had continuous access to HCQ water starting 24 h prior to tumor cell injection. HCQ water was replaced every three days. Regular drinking water was given to control mice. Verdiperstat (MedChemExpress #HY-17646), a known MPO inhibitor, was given via daily intraperitoneal injection at 1.37 mg in 300 µL. Verdiperstat was made by initially dissolving powder in 100% DMSO. This was then diluted by adding hydroxypropyl-β-cyclodextrin (MedChemExpress, #HY-101103) to a final solution of 4% DMSO. Verdiperstat was administered daily until euthanasia, which was performed after 2–3 weeks of treatment unless it was administered as part of a survival study, in which case it was administered until humane endpoints were met.

### Metastatic PDAC models

A C57Bl/J6 murine pancreatic adenocarcinoma cell line, Pan02 cells (National Cancer Institute Repository, 2008), was used in our experiments. Cells were cultured in a humidified incubator in 5% CO_2_ using RPMI (Fisher, EH30255FS) with 10% fetal bovine serum (FBS) (Fisher, MT35010CV) and 1% penicillin-streptomycin (P/S) (Gibco, #15-140-148). For the liver metastases model, we first anesthetized WT or PAD4^−/−^ mice aged 8 to 14 weeks using 90 mg/kg intraperitoneal ketamine, 10 mg/kg intraperitoneal xylazine as well as 0.1 mg/kg subcutaneous buprenorphine for pain control. The portal vein was identified after a midline laparotomy. We used a Hamilton syringe to inject 1 × 10^6^ cells in 10 µL PBS into the portal vein, which leads directly to the liver. For the peritoneal metastasis model, 1 × 10^6^ Pan02 cells were injected into the peritoneum. Mice were euthanized between 2 and 3 weeks later.

### Murine survival studies

Liver metastases were established via portal vein injection of 0.5 × 10^6^ cells as described above. A nine-point pain and distress physiologic scoring system was utilized to monitor the clinical status of mice and ensure humane endpoints were utilized. Mice were evaluated by a lab member blinded to treatment group to avoid bias.

### Murine neutrophil isolation

Murine neutrophils were collected from the bone marrow of femurs of WT mice. After cutting the ends of the femurs, bone marrow was rinsed with 10 mL RPMI containing 10% FBS and 1% P/S (complete media) over a 100 μm nylon mesh filter atop a 50-mL conical tube. The solution was centrifuged at 1400 rpm for 7 min at 4 °C, washed with 10 mL complete media, and centrifuged again. Cells were separated using density gradient centrifugation where 3 mL room temperature (RT) Histopaque 1077 (Millipore Sigma, #10771) were layered over 3 mL RT Histopaque 1119 (Millipore Sigma, #11191). Cell solution in 1 mL PBS was added last followed by centrifuging for 30 min at 2000 RPM 25 °C without a break. The layer between Histopaque 1077 and 1119 containing neutrophils was collected. Cells were washed twice with RPMI and centrifuged for 7 min at 1400 RPM 4 °C. Cells were resuspended at 1 × 10^5^ cells per 100 µL and plated in a 96 black-walled plate containing PBS control, HCQ diluted in PBS, or Verdiperstat. Verdiperstat was initially dissolved in 100% DMSO. Once added to cell solution, though, the final concentration of DMSO per well was 1%. Neutrophils were incubated with treatment at 37 °C in a humidified incubator for 30 min. Phorbol myristate acetate (PMA) (Sigma Aldrich, #P8139) and luminol sodium salt (Sigma Aldrich, #20666-12-0) were then added for a final concentration of 500 nM and 50 mM, respectively. Cells were immediately imaged at 37° C with 5% CO_2_ for 60 min on the Kino imaging system (Spectral Instruments Imaging, AZ, USA). ROI measurements were performed using the Aura analysis software (Spectral Instruments Imaging, AZ, USA). The four replicates from each time point were averaged and used to calculate the area under the curve.

### Circulating NET markers

Blood was collected from mice via cardiac puncture. Blood was centrifuged for 10 min at 1000 RPM, and plasma was collected. After diluting the plasma at 1:10, the QuanTi PicoGreen dsDNA reagent kit (Fisher, #P11495) was used to measure plasma cell free DNA (cfDNA) (Fisher, #P11495) according to the manufacturer’s protocol.

### Ex vivo bioluminescence imaging

Luminol sodium salt was reconstituted at 50 mg/mL in PBS. For ex vivo imaging, mice were given an intraperitoneal injection of 100 µL (5 mg) luminol. The liver was removed, and bioluminescence imaging was performed on the IVIS Spectrum bioluminescence imaging system (Perkin Elmer/Caliper Life Sciences, MA, USA) 10 min after luminol injection. The Living Imaging analysis software (Spectral Instruments Imaging, AZ, USA) was used to measure ROI and then area under the curve was calculated.

### Immunohistochemistry

Liver tumor was collected and immediately placed in formalin for fixation. After 24–48 h tissue was transferred to 70% ethanol. Histology was performed at Histowiz Inc (NY, USA) using a Standard Operating Procedure and fully automated workflow. Samples were processed, embedded in paraffin, and sectioned at 4 μm. IHC was performed on Leica Bond RX automated stainer (Leica Microsystems). The slides were dewaxed using xylene and alcohol based dewaxing solutions. Epitope retrieval was performed by heat-induced epitope retrieval (HIER) of the formalin-fixed, paraffin-embedded tissue using citrate-based pH 6 solution for 20 min. The slides were then incubated with one of the following antibodies for 80 min: CD8a antibody (Cell signaling technology, cst85336) at 1:200 dilution, Ly6g (Gr1) 6c (mouse) antibody (Abcam, ab25377) at 1:300 dilution, Myeloperoxidase (MPO) (hu ms) (Abcam, ab9535) at 1:50 dilution. After, DAB rabbit secondary reagents (polymer, DAB refine and hematoxylin (Bond Polymer Refine Detection Kit, Leica Microsystems)) were applied according to the manufacturer’s protocol. The slides were dried, cover slipped (Tissue-Tek Prisma Coverslipper), and scanned using a Leica Aperio AT2 slide scanner at 40X.

### Molecular modeling and docking studies

The structure of HCQ was downloaded from PubChem (ID:3652) as a 3D conformer and verified using VIDA 5.0.5.3 (Open Eye). For the docking studies, MOE2022.02 (Chemical Computing Group) was used with the structure of MPO (7NI1.pdb). The protein structure was imported and prepared for docking by adding hydrogens, fixing missing side chains, and running a quick energy minimization to remove steric interactions with the pH of the system set at 7.4 to add partial charges. For the docking simulation, the co-crystal structure, CPD 9, was identified as a ligand which delineated the binding pocket. Placement of hydroxychloroquine in the binding pocket for docking used the Triangle Matcher method using the London dG score, returning 30 poses, followed by refinement with the Induced Fit method, using a GBVI/WSA dG scoring system.

### Surface Plasmon Resonance (SPR)

SPR studies were performed by Creative Biolabs (New York USA). Human MPO (R&D, #3174-MP-250) w*as* attached to a CM5 sensor chip using standard amine coupling reagents and protocols. A dose-response interaction between hydroxychloroquine and MPO was analyzed in a Biacore 1 K. From the sonograms, the equilibrium dissociation constant Kd was determined for both proteins.

### Human samples and human neutrophil isolation

Patients diagnosed with PDAC at a single tertiary care center were identified and consented for blood procurement (IRB #2103260669). Blood was collected following completion of neoadjuvant chemotherapy, most often immediately preoperatively. Patients from our clinical trial (NCT04911816) evaluating HCQ combined with chemotherapy in the perioperative setting were included. Inclusion criteria were as follows: biopsy-proved PDAC, pancreatic protocol helical CT scan demonstrating resectable disease consistent with NCCN guidelines, ECOG performance status of at least one, no active second malignancy, normal renal, hepatic, and hematologic function, serum creatinine within 1.5 the upper limits of normal, serum total bilirubin within 1.5 the upper limits of normal, white blood cell count over 3.5 × 10^9^/mL, and platelet count 100 × 10^9^/mL. Exclusion criteria were as follows: chemotherapy within 12 months prior to study entry, loss-of-function mutations in DPYD or UGTA1, prior use of radiotherapy or investigational agents for PDAC, and borderline resectable, locally advanced or metastatic disease. Eligible patients received HCQ twice daily (total 400 to 800 mg daily) in addition to their neoadjuvant chemotherapy. HCQ treatment was started the same day as neoadjuvant chemotherapy and continued until two weeks postoperatively. Analysis was performed from isolated neutrophils after 6 to 8 weeks of HCQ treatment (while patients remained on HCQ) and after 4 cycles of chemotherapy. Blood samples obtained from control patients who were not enrolled in the trial and received 4 cycles of chemotherapy were obtained from the WVU Biospecimen and Translational Research Analysis Core (BioTRAC) in a de-identified fashion.

Human neutrophils were isolated according to the manufacturer’s protocol (MACSxpress Whole Blood Neutrophil Isolation Kit; Miltenyi Biotec, Waltham, MA, USA). Neutrophils (1 × 10^5^) were added to 50mM luminol in 100uL PBS in a 96-well black-walled plate. Neutrophils were immediately imaged for 30–60 min using the IVIS Spectrum bioluminescence imaging system (Perkin Elmer, MA, USA) or the Kino imaging system (Spectral Instruments Imaging, AZ, USA) at 37ºC under 5% CO2 flow. The typical acquisition parameters were acquisition time (autoexposure), binning (8), field of view (FOV: 15 cm), f/stop (1), filter (open), image-image interval (5 min), and total number of acquisitions (6 to 12). Bioluminescence photon flux (photons/s) data were analyzed by region of interest measurements with background subtraction in Living Image 4.5 (Perkin Elmer, Waltham, MA, USA) or Aura (Spectral Instruments Imaging, AZ, USA). These raw data were imported into Excel (Microsoft Corp, Redmond, WA, USA) and averaged in each individual experiment performed in triplicate wells. The area under the curve was calculated from images taken for 30 to 60 min.

### Statistics

Statistical analyses were performed using GraphPad Prism 10 (Version 10.2.2). Murine data were analyzed using unpaired student’s two-tailed t-tests. Human data were analyzed using a Mann-Whitney test for continuous data or a Fisher’s exact test for categorical. Statistical significance was defined as *p* < 0.05.

## Results

### HCQ reduces metastatic PDAC tumor burden and improves survival in a NET-independent manner

**Fig. 1:**
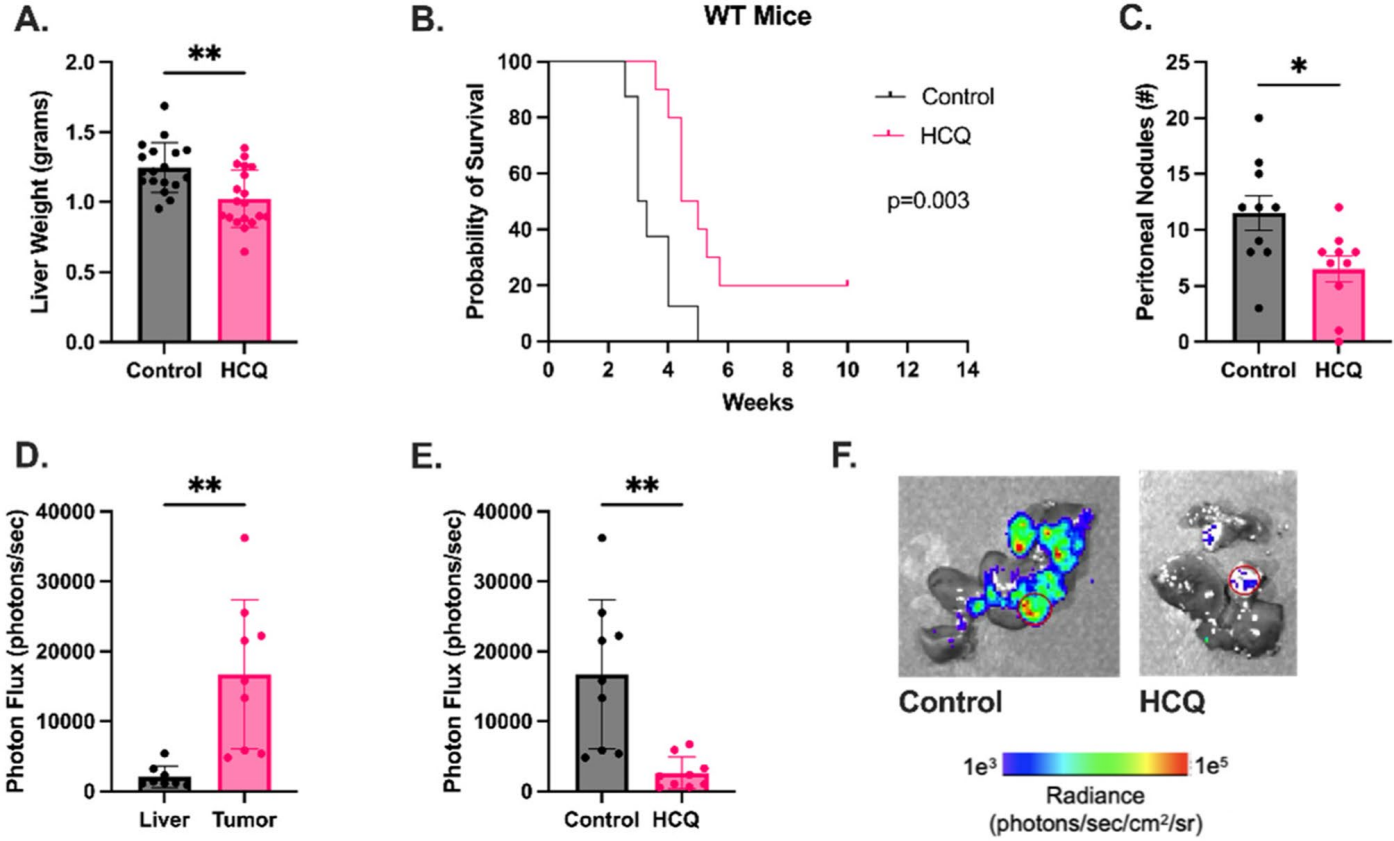
Hydroxychloroquine reduces metastatic tumor burden in murine PDAC. **A** HCQ treatment in WT mice significantly reduced liver tumor burden and (**B**) improved survival using humane endpoints. **C** Peritoneal tumor burden was also significantly decreased with HCQ treatment compared to control. **D** Liver tumors had increased luminol bioluminescence, reflective of MPO activity, compared to adjacent liver (**E**) which was reduced with HCQ treatment. **F** Representative luminol bioluminescence images. * = *p*-value < 0.05; ** = *p*-value < 0.01

Given the capacity of HCQ to inhibit NETs and the established role of NETs in the formation of metastases, we sought to investigate the role of HCQ pre-treatment as a means to limit PDAC. We utilized two metastatic models that replicated the common sites of PDAC metastasis. First, we injected Pan02 cells into the portal vein, leading to growth of liver lesions. We found treatment with HCQ starting 24 h prior to tumor cell injection resulted in significantly decreased tumor burden (Fig. 1A, 1.0 g versus 1.3 g; *p* = 0.008). Given these impressive results, we performed a survival study in wild type (WT) mice treated with HCQ. Mice were evaluated for humane endpoints blinded to the treatment group. HCQ treated mice had a significantly prolonged survival compared to control (Fig. 1B and 4.7 versus 3.1 weeks; *p* = 0.003). Similarly, intraperitoneal injections of Pan02 cells led to the development of peritoneal nodules. The total number of peritoneal nodules was also significantly decreased with HCQ treatment (Fig. 1C and 6.5 versus 11.5 nodules; *p* = 0.02). Given HCQ’s ability to inhibit the enzyme PAD4, and subsequent NET formation, we hypothesized the antineoplastic effects of HCQ were due to decreased NETosis. We utilized luminol bioluminescence imaging to measure MPO. Although MPO is not specific to NETs, it is expressed within NETs and is involved in stimuli-specific NET formation through production of reactive oxygen species [41, 42]. In luminol bioluminescence imaging, luminol is oxidized via ROS produced by MPO resulting in the release of blue luminescence [43]. This specialized imaging has been previously validated by authors of this study and is an established measure of MPO activity in the literature [44, 45]. Ex vivo liver tumors were found to have an increase in MPO activity compared to normal liver tissue (Fig. 1D, 1.67 × 10^4^ versus 2.08 × 10^3^ photons/second; *p* = 0.002). HCQ significantly reduced this activity (Fig. 1E-F and 2.62 × 10^3^ versus 1.67 × 10^4^; *p* = 0.001). Although MPO can be used as a marker of NETs, its activity is not isolated to NETs. Therefore, we measured plasma cell free DNA (cfDNA), another validated marker of NETs in murine PDAC [46]. However, there was no difference between WT mice treated with HCQ compared to control (Supplementary Fig. 1A). Additionally, we found liver tumor burden in PAD4^−/−^ mice was similar to WT control, regardless of HCQ treatment (Supplementary Fig. 1B). These data suggest a NET independent anti-tumor mechanism from HCQ treatment in these PDAC metastatic disease models.

### HCQ inhibits MPO activity

**Fig. 2:**
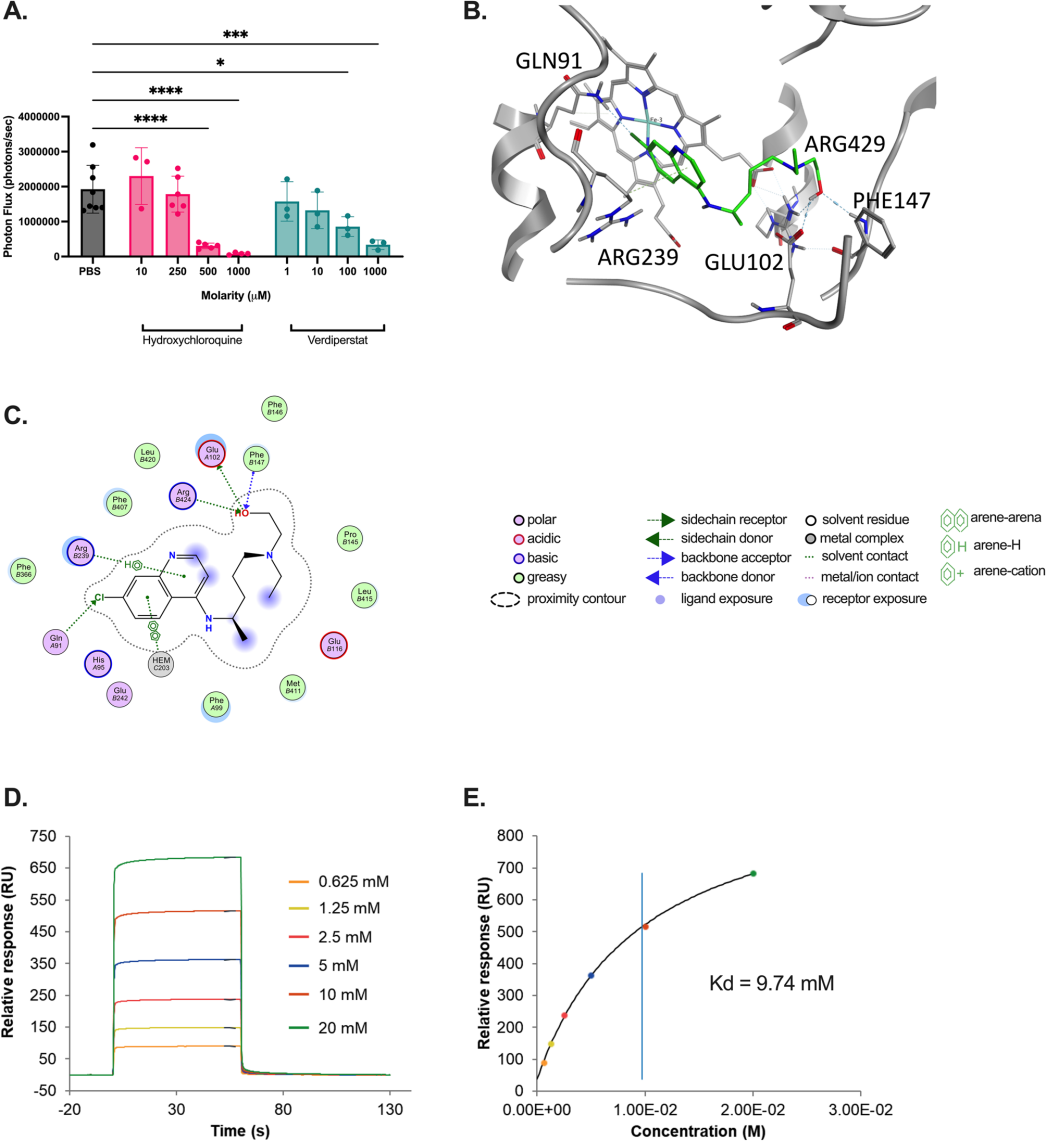
Hydroxychloroquine inhibits myeloperoxidase activity in vitro. **A** HCQ inhibited in vitro MPO function similar to a known selective MPO inhibitor, verdiperstat, in isolated neutrophils. **B**-**C** Molecular docking studies of HCQ in MPO (7NI1.pdb) demonstrated HCQ localizing close to the heme group of MPO, blocking substrate access. Major amino acid interactions are shown. **D**-**E** SPR analysis determining binding potential of HCQ to MPO with an equilibrium dissociation constant (Kd) of 9.74 mM. * = *p*-value < 0.05; ** = *p*-value < 0.01; *** = *p*-value < 0.001

We hypothesized the HCQ-mediated difference in MPO activity was independent of NET formation. To test this hypothesis, we isolated neutrophils from WT mice and quantified MPO function in the presence of control, HCQ, or a known selective, irreversible MPO inhibitor, verdiperstat. Both HCQ and verdiperstat inhibited MPO enzymatic function in vitro (Fig. 2A, *p* **< **0.0001). Despite the numerous known mechanisms of action of HCQ, MPO inhibition has not previously been described. Therefore, we performed computational modeling of the interaction between HCQ and MPO to determine potential binding sites. Interaction analysis identified a hydrogen bond interaction between HCQ and Gln91, Arg 239, Arg429, Glu102, and Phe147 residues of MPO (Fig. 2B-C). SPR analysis then confirmed the interaction between HCQ and human MPO with an equilibrium dissociation constant (Kd) of 9.74 mM (Fig. 2D-E).

### MPO inhibition by HCQ in vivo in metastatic murine PDAC

**Fig. 3:**
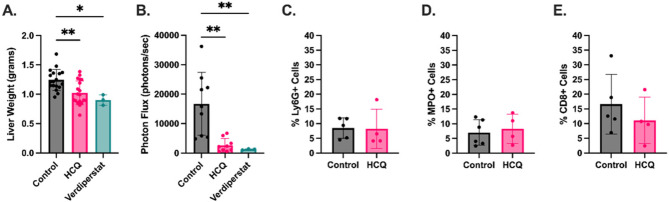
Myeloperoxidase inhibition by hydroxychloroquine prolongs survival in vivo. **A** HCQ reduces tumor burden and (**B**) intratumoral bioluminescence similar to Verdiperstat, an MPO inhibitor. **C** HCQ does not impact neutrophil infiltration or (**D**) intratumoral MPO expression, indicating HCQ inhibits the enzymatic activity of MPO and does not alter its production. **E** HCQ treatment did not impact CD8 + cell infiltration in liver metastases. * = *p*-value < 0.05; ** = *p*-value < 0.01

With these findings, we returned to our murine model to further understand the implications of this interaction between HCQ and MPO. Given the many mechanisms of action of HCQ, we sought to confirm the tumor burden reduction was truly secondary to MPO inhibition. We found treatment with MPO inhibition via verdiperstat had a similar effect on tumor burden (Fig. 3A, 0.90 g after verdiperstat versus 1.26 g with control; *p* = 0.04) and intratumoral luminol bioluminescence as HCQ, reflective of MPO activity (Fig. 3B 1.24 × 10^3^ after verdiperstat versus 1.67 × 10^4^ with control; *p* = 0.01). While verdiperstat is a selective MPO inhibitor and has no known off target effects, we also sought to confirm the decreased MPO activity was not simply due to a lack of MPO in the tumor microenvironment. While MPO can be expressed in monocytes at low levels, the vast majority of MPO resides in the granules of neutrophils [47, 48]. We confirmed neutrophil infiltration was similar between control and HCQ treated tumors via Ly6G IHC staining (Fig. 3C). MPO expression and production within neutrophils is determined early in neutrophil development. Once neutrophils exit the bone marrow, no further MPO is produced [46]. MPO staining of tumors showed similar MPO expression between tumors, further supporting the hypothesis that HCQ inhibits the enzymatic activity of MPO as opposed to the production or expression of MPO or recruitment of MPO containing cells (Fig. 3D). MPO can contribute to the immune landscape within the tumor microenvironment, so we quantified intratumoral CD8 + cell infiltration [23, 44, 45, 49, 50]. We found no difference in T cell infiltration with HCQ treatment (Fig. 3E).

### HCQ inhibits MPO activity in human PDAC patients

**Fig. 4:**
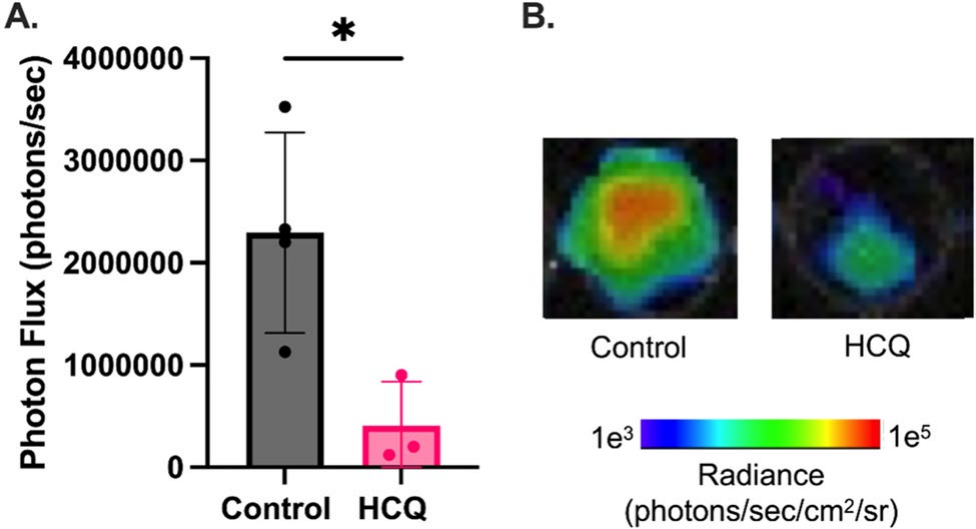
Hydroxychloroquine inhibits MPO function in patients with PDAC. **A** Neutrophils isolated from HCQ treated PDAC patients demonstrated decreased luminol bioluminescence, reflective of MPO activity compared to control patients treated with chemotherapy alone (no HCQ). **B** Representative luminol bioluminescence images. * = *p*-value < 0.05

We next accessed MPO function in patients with PDAC following neoadjuvant chemotherapy. As part of an ongoing clinical trial at our institution (NCT04911816), three patients had received oral HCQ twice daily (400–800 mg total) as a component of their neoadjuvant treatment. These patients were compared to patients who received neoadjuvant chemotherapy alone without prior history of HCQ use. Patient characteristics are compared in Table 1, demonstrating no significant differences between groups. Circulating neutrophils from post-treatment blood samples were isolated and MPO activity quantified using luminol bioluminescence. HCQ treatment resulted in a reduction in MPO activity, confirming this novel mechanism of action in PDAC patients (Fig. 4, 4.08 × 10^5^ versus 2.30 × 10^6^; *p* = 0.03).

**Table 1:** Patient characteristics

	Chemotherapy Alone *N* = 4	Chemotherapy + Hydroxychloroquine *N* = 3	*p* value
Age, mean (SD)	53.0 (12.3)	68.3 (4.6)	0.10
Female, n (%)	3.0 (75.0)	1.0 (33.3)	0.49
Stage, n (%)			0.99
I	2.0 (50.0)	1 (33)	
II	2.0 (50.0)	2 (67)	
Chemotherapy Regimen, n (%)			0.43
FOLFIRINOX	2.0 (50.0)	2.0 (66.7)	
Gemcitabine and Nab-paclitaxel	2.0 (50.0)	0.0 (0.0)	
Combination	0.0 (0.0)	1.0 (33.3)	
Chemotherapy Cycles, mean (SD)	5.5 (2.6)	4.3 (1.5)	0.53

## Discussion

In this study we identified a new mechanism of action of HCQ and its potential impact in metastatic PDAC. Although there are limited PDAC trials involving HCQ, there is evidence of good tolerability when given in combination with gemcitabine or gemcitabine nab-paclitaxel treatment for all stages, even at the maximum tested dose of 1200 mg per day [51, 52]. In potentially resectable disease, the addition of HCQ to neoadjuvant gemcitabine and nab-paclitaxel was associated with a higher histopathological response rate and Ca 19 − 9 response [39]. A small study of 35 patients demonstrated encouraging improvements in survival after neoadjuvant HCQ and gemcitabine in patients with resectable disease [38]. Only two studies have evaluated HCQ in the setting of metastatic PDAC. First in 2014, 20 metastatic patients were given either 400 mg or 600 mg HCQ twice daily as a monotherapy after failing one (30%) or two (70%) prior treatment regimens [53]. There was no difference in the primary endpoint of two-month progression free survival. While these results could be interpreted as failing to support HCQ as a monotherapy, this small trial focused on PDAC patients with particularly advanced metastatic disease. This study’s outcomes are difficult to compare to other trials, which often included untreated metastatic patients or those who have failed a single regimen [54]. In 2019, untreated metastatic or locally advanced PDAC patients were randomized to gemcitabine and nab-paclitaxel with or without HCQ with no difference seen in OS at one year [40]. However, there were improvements in treatment response rates in HCQ treated patients. While half of the metastatic patients in each group had liver disease, there was an unequal distribution of lung and peritoneal disease between groups. Given the prognostic differences between metastatic sites, tumor biology likely also differs by location, and therefore, so may treatment responses [5, 55]. The current study provides support for HCQ treatment of liver metastases, particularly when given early in the disease course. Additionally, since HCQ treatment was initiated shortly prior to tumor inoculation in our study, HCQ may be able to prevent the formation of metastases. This, in combination with its favorable side effect profile, highlights that HCQ should be further investigated as a chemopreventative agent.

In our study, we found a significant reduction in liver tumor burden as well as prolonged murine survival with HCQ treatment. We originally hypothesized this was mediated by NET inhibition via HCQ blockade of PAD4. However, we found genetic NET inhibition via PAD4 deficiency resulted in similar tumor burden to untreated WT mice. Interestingly, the effect of HCQ also disappeared in PAD4^*−/−*^ mice. There is conflicting data linking NETs and metastases. In some contexts, NETs can exert anti-neoplastic effects [50, 56]. In fact, Takesue et al. found NET inhibition via DNase treatment did not influence PDAC liver metastases in a spontaneous mouse model [57]. However, the vast majority of data implicate NETs in tumor progression and the promotion of metastases [24, 58–60]. Similarly, PAD4 has largely been described in regards to its role in NETosis and subsequent tumor progression. However, through protein citrullination, PAD4 has many downstream effects on gene expression and protein function [59]. Protein citrullination by PAD4 also antagonizes DNA methylation, further impacting gene expression [61]. These modifications often promote tumor growth and metastasis, but varying results have been shown in liver malignancies. Zhang et al. demonstrated that many patients with a primary liver malignancy have lower intra-tumoral PAD4 expression than surrounding tissue and that those with high intra-tumoral PAD4 expression had prolonged survival [62]. This is in contrast to Yuzhalin et al., who showed that the effects of PAD4 promoted the growth of colorectal cancer liver metastases [63]. These dissimilarities may indicate a different role for PAD4 depending on the clinical context, which is not unexpected given the variety of downstream proteins it affects.

After identifying the benefit of HCQ treatment was independent of NETs, we discovered a new potential target of HCQ, the enzyme MPO. In addition to contributing to neutrophil recruitment, MPO catalyzes a reaction between hydrogen peroxide and halides, resulting in the production of ROS [39]. These ROS are important for microbial clearance but can also lead to oncogenic mutations that promote carcinogenesis and tumor growth [64]. While cancer cell apoptosis can be induced as a result of ROS, cancer cells adapt through protective pathways, such as increased expression of membrane catalases [65, 66]. MPO-derived oxidants also play a role in extracellular matrix remodeling to promote metastasis [47, 67]. The role of MPO in adaptive immunity is complex and appears context dependent. Some authors report enhanced CD8 infiltration or antigen presentation as a result of MPO function, while others find MPO limits CD8 infiltration [44, 47]. Dendritic cell function and infiltration appears to be negatively impacted by MPO [46, 68]. Liu et al. found MPO activity was increased in the setting of melanoma, resulting in increased myeloid cell populations both within tumors and systemically. Inhibition of MPO resulted in increased CD8 infiltration and prolonged survival as well as enhanced response rates to immunotherapy [45]. Improved immunotherapy responses after MPO inhibition have also been demonstrated in primary PDAC with associated changes in the intratumoral immune landscape [23]. Lastly, MPO’s positive charge allows it to bind and modify the structure and functionality of multiple proteins [69, 70]. However, this aspect of MPO functionality has not been investigated in the setting of cancer. In this murine model of metastatic PDAC, we found intratumoral MPO activity was upregulated compared to adjacent tissue. MPO inhibition resulted in decreased tumor burden and prolonged survival. While there was no difference in CD8 infiltration, intra-tumoral ROS production was significantly reduced. Given the known downstream effects of MPO-derived oxidants on ECM modulation, this mechanism for anti-metastatic effects of MPO inhibition warrants further investigation in future studies.

To our knowledge, we are the first to identify HCQ’s ability to inhibit MPO function. Even in humans, where MPO levels are 5 to 10 times that of mice, we found HCQ could effectively block MPO function at doses that have demonstrated safety and good tolerability in patients receiving chemotherapy [71]. While there are multiple specific MPO inhibitors under investigation, there is currently no FDA approved agent. Therefore, the addition of HCQ as an MPO inhibitor fills a significant need in cancer care. Of course, success in murine models does not always translate to success in human patients. However, the preliminary data from our clinical trial provides hope that long-term outcomes such as response rates and survival will reflect the role of MPO inhibition via HCQ therapy in PDAC patients.

While this work highlights an important use for HCQ, it has limitations. First, it is difficult to simulate metastases in a murine model. While our portal vein injection method and the splenic injection method are commonly used, there is no primary tumor in the model to prime the pre-metastatic niche. These results must be interpreted in this context. In this study, HCQ was given as a pre-treatment prior to tumor injection. Based on the findings in the current study, HCQ treatment may have applications in limiting metastatic disease in the neoadjuvant setting or in patients with localized disease who are not surgical candidates. Additionally, we attribute differences in luminol bioluminescence to be secondary to MPO activity, consistent with established literature [43–45, 72]. However, some in vitro studies show luminol can be oxidized by hydrogen peroxide (H_2_O_2_), independent of MPO [43]. Conversely, Gross et al. showed in the setting of an MPO inhibitor, H_2_O_2_ was unable to produce bioluminescence by luminol [43, 73]. For this reason, we also incorporated the MPO inhibitor, verdiperstat, into our studies and showed similar reductions in bioluminescence both in vitro and in vivo. Lastly, we do not confirm the mechanism by which MPO inhibition delivers its anti-neoplastic properties. As previously discussed, ROS accumulation impacts many downstream pathways. The effect could be a combination of these or in relation to the immune modulating effects of MPO. While we saw no difference in CD8 infiltration, we did not test the functionality of these cells or evaluate the presence of immunosuppressive cells. These are all goals of our future work.

## Conclusions

In summary, we describe a new mechanism of HCQ, a drug that has a well-studied safety profile, low cost, and oral bioavailability. MPO inhibition through HCQ resulted in decreased tumor burden and prolonged survival in a murine model of metastatic PDAC. Furthermore, we established HCQ’s ability to inhibit MPO function in human PDAC patients. This work demonstrates HCQ as a potential treatment option for metastatic PDAC and should be investigated further in clinical trials.

## Supplementary Information

Supplementary Material 1: Supplementary Figure 1. Relationship between HCQ anti-neoplastic effect and NETs. (A) Plasma cell free DNA in WT mice is similar after treatment with control or HCQ. (B) HCQ reduces tumor burden in WT mice but has no effect in PAD4-/- mice. Additionally, *PAD4*^*-/-*^mice have tumor burdens comparable to control treated WT mice. * = *p*-value < 0.05.

## Data Availability

Data is provided within the manuscript or supplementary information files. Raw data can be provided upon request.

## Abbreviations

cfDNA: Cell free DNA
FBS: Fetal bovine serum
HCQ: Hydroxychloroquine
MPO: Myeloperoxidase
NE: Neutrophil elastase
NETs: Neutrophil extracellular traps
OS: Overall survival
P/S: Penicillin-streptomycin
PAD4: Peptidyl arginine deiminase 4
PAD4-/-: Peptidyl arginine deiminase 4 knockout
PDAC: Pancreatic adenocarcinoma
PMA: Phorbol myristate acetate
ROS: Reactive oxygen species
RT: Room temperature
SPR: Surface plasma resonance
WT: Wild type
