# Supplementary material for: Hydroxychloroquine reduces metastatic tumor burden in pancreatic adenocarcinoma through myeloperoxidase inhibition
Source: J Inflamm (Lond). 2025 Aug 12;22:33. doi: 10.1186/s12950-025-00456-8 (PMC12344839; doi:10.1186/s12950-025-00456-8)
Supplement: Supplementary file 1 — Supplementary Material 1: Supplementary Figure 1. Relationship between HCQ anti-neoplastic effect and NETs. (A) Plasma cell free DNA in WT mice is similar after treatment with control or HCQ. (B) HCQ reduces tumor burden in WT mice but has no effect in PAD4-/- mice. Additionally, PAD4-/-mice have tumor burdens comparable to control treated WT mice. * = p-value < 0.05. [file 12950_2025_456_MOESM1_ESM.docx]

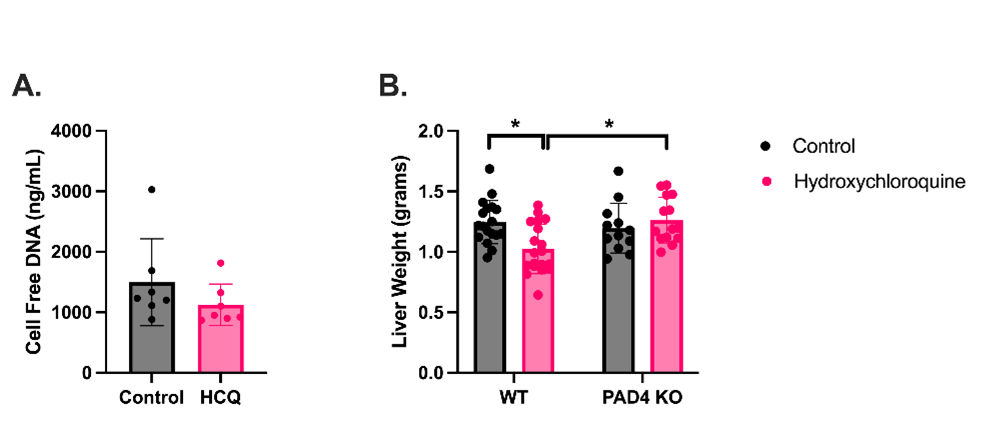


**Supplementary Figure 1. Relationship between HCQ anti-neoplastic effect and NETs.**

(A) Plasma cell free DNA in WT mice is similar after treatment with control or HCQ. (B) HCQ reduces tumor burden in WT mice but has no effect in PAD4-/- mice. Additionally, *PAD4^-/-^* mice have tumor burdens comparable to control treated WT mice. * = p-value < 0.05.
